# An Overview of Key Technologies in Physical Layer Security

**DOI:** 10.3390/e22111261

**Published:** 2020-11-06

**Authors:** Abraham Sanenga, Galefang Allycan Mapunda, Tshepiso Merapelo Ludo Jacob, Leatile Marata, Bokamoso Basutli, Joseph Monamati Chuma

**Affiliations:** 1 Electrical, Computer, and Telecommunications Engineering, Botswana International University of Science and Technology, Palapye, Botswana; galefang.mapunda@studentmail.biust.ac.bw (G.A.M.); tshepiso.jacob@studentmail.biust.ac.bw (T.M.L.J.); basutlib@biust.ac.bw (B.B.); chumaj@biust.ac.bw (J.M.C.); 2 Center for Wireless Communications, University of Oulu, 90570 Oulu, Finland; maratal@biust.ac.bw

**Keywords:** artificial noise, beamforming, intelligent reflective surface, MIMO, optimization, physical layer security, zero forcing

## Abstract

The open nature of radio propagation enables ubiquitous wireless communication. This allows for seamless data transmission. However, unauthorized users may pose a threat to the security of the data being transmitted to authorized users. This gives rise to network vulnerabilities such as hacking, eavesdropping, and jamming of the transmitted information. Physical layer security (PLS) has been identified as one of the promising security approaches to safeguard the transmission from eavesdroppers in a wireless network. It is an alternative to the computationally demanding and complex cryptographic algorithms and techniques. PLS has continually received exponential research interest owing to the possibility of exploiting the characteristics of the wireless channel. One of the main characteristics includes the random nature of the transmission channel. The aforesaid nature makes it possible for confidential and authentic signal transmission between the sender and the receiver in the physical layer. We start by introducing the basic theories of PLS, including the wiretap channel, information-theoretic security, and a brief discussion of the cryptography security technique. Furthermore, an overview of multiple-input multiple-output (MIMO) communication is provided. The main focus of our review is based on the existing key-less PLS optimization techniques, their limitations, and challenges. The paper also looks into the promising key research areas in addressing these shortfalls. Lastly, a comprehensive overview of some of the recent PLS research in 5G and 6G technologies of wireless communication networks is provided.

## 1. Introduction

Wireless communication technology is a necessity for modern-day life because human beings depend on this technology for data transmission. In most cases, the said data contain confidential information such as banking transactions, military applications, and multimedia. The International telecommunication organization approximated that 53.6% of the world population, which amounts to about 4.1 billion people, were using the internet at the end of 2019 [1]. It is expected that this number will rise due to a rapid increase in active mobile subscribers as wireless networks continue to expand and new applications are developed. However, it is reported in [2] that an increasing number of mobile and wireless devices are affected by cyber-criminal activities. Cyber security ventures [2] predicted that cyber crime costs will increase to more than 6 trillion US dollars annually by 2021, from 3 trillion US dollars recorded in 2015. Therefore, it is highly important to improve wireless networks against cyber-criminal activities.

Conventionally, the upper layers of the open system interconnect model are utilized to handle any discrepancies related to the attributes of authenticity, confidentiality, and privacy of data transmission. These attributes are mostly dependent on cryptographic algorithms which include secret-key distribution, public-key, and symmetric encryption. All these techniques function independently from the physical layer [3]. Based on the assumption that the eavesdropper has limited computing power ability, the above-mentioned techniques are considered to be secure. Moreover, they rely on underlying computational complexity for their robustness. Recent advances in quantum computing pose a serious threat to the currently used cryptographic schemes with their unlimited computational capacity [4]. Therefore, it is evident that the conventional methods in secure wireless communication are becoming less reliable.

The open and superposition nature of wireless networks raises issues of confidentiality and security of the transmitted data when unintended users are present. Difficulties that prevent the transmitted signal from reaching the unintended users are a result of the broadcast nature. On the other hand, time variations and fluctuations in the wireless channel result in the arrival of multiple copies of the transmitted signal at the receiver. With that being said, security attacks in wireless networks may be categorized as active and passive attacks [5]. Passive attacks involve eavesdroppers who listen to the ongoing transmission silently and try to steal the transmitted information without interrupting legitimate transmission [6]. Under active attacks, the eavesdroppers use more aggressive and intrusive techniques that attempt to deteriorate the quality of the signal at the intended receiver. Common examples of these aggressive techniques are the denial of service, routing, and node malfunction attacks [5]. With exposure to so many attacks, wireless networks are required to have certain capabilities that will enable them to withstand and mitigate these attacks. The desired characteristics of a secure network include integrity, confidentiality and authentication, availability, and access control [5].

### 1.1. Related Works

In this section, we provide a brief overview of some of the related works on the review of key technologies in PLS. It should be noted that this paper focuses only on key-less PLS technologies. The survey paper in [7] provides a comprehensive overview of security in the physical layer from both an information-theoretic point of view and optimization using multi-antenna techniques; however, it does not describe applications of PLS techniques in modern wireless communication networks. To the best of our knowledge, the reviews found in [8,9] are the only ones that cover the application of conventional PLS techniques in 5G (and beyond) wireless communication systems. They give insight into some of the emerging technologies, including internet of things (IoT), multiple-input multiple-output (MIMO), energy harvesting, visible light communication (VLC), and UAV communication. However, there are a lot of papers available that focus on individual aspects of the technologies mentioned above.

VLC: Recently, the authors in [10] provided a comprehensive survey of VLC applications in PLS. The paper also discusses VLC channel models, different network configurations, and also presents some of the precoding strategies. In this paper, however, we demonstrate, with some simulation plots, a VLC practical scenario model with realistic system design parameters and show how the optical power decay in VLC is beneficial in PLS.

IRS: Another technology which has gained a lot of interest in PLS recently is intelligent reflecting surfaces, and there has been a lot of work published in this area. Some recent papers are [11,12,13]. The authors in [11] provide an overview of IRS technology, particularly its applications across the whole of wireless communication. They also outline the advantages IRS offers and some of the challenges in implementing IRS-aided systems. The optimization problems in the IRS-based PLS scheme are derived and solved in [12,14]. In this paper we specifically demonstrate the application of IRS in PLS and evaluate the performance of an IRS-based scheme using the achievable secrecy rate metric.

UAVs: There have been a couple of papers published in this area recently [15,16,17]. Current research is aimed at the integration of UAV communication networks with 5G technology (and beyond 5G) to safeguard the current and future wireless networks. We provide a brief overview and summary of some of the interesting works on the applications of UAVs in PLS.

Satellite communication: This is another area that has become a trend in recent PLS research, and a couple of interesting works have been published in [18,19,20,21]. Vasquez et al. [18] provided an overview of precoding techniques in multibeam satellite communication systems. Another study [19] proposed the inter-satellite communication of small satellite systems. A comprehensive overview of PLS in space information networks is given in [21]. They proposed the integration of satellite and IoT to form a satellite-based IoT and also discussed current technologies dedicated to PLS in land mobile satellite communication networks. Our letter intends to summarize some of the promising technologies that are discussed in the literature.

### 1.2. Overview of Cryptography and PLS

**Figure 1:**
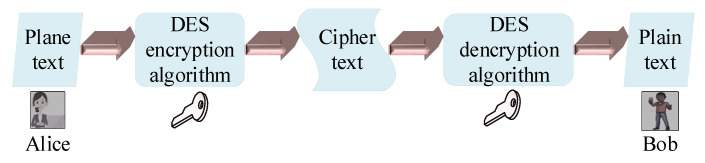
The process of cryptography [23].

Cryptography is the method of transforming data into an unreadable format so that only the authorized recipient can understand and be able to decode it [22]. The main process of cryptography is shown in Figure 1. Encryption uses coding to transform plain text into an unreadable format, whereas decryption uses a decoding process to convert the unreadable text to a piece of readable information using some special keys. Cryptography can be divided into three main types: hash functions, public key cryptography, and secret key cryptography. The latter uses only one digital key to encrypt and decrypt data for both the sender and the receiver. Meanwhile, the first mentioned type utilizes a pair of public digital keys. In this case, one key is a public key used by the sender to encrypt the message, and the other key is a secret key used by the receiver to decrypt the information. Lastly, hash functions are a type of cryptography method that uses a hash value of a fixed length encrypted into the plain text. Hash functions use algorithms to generate a digital fingerprint to create one-way encryption [22].

In most cases, cryptography is the main technology utilized to address security issues for conventional and some modern-day electronic communication systems. From another perspective, some novel technologies, mainly quantum computing, are a threat to systems that are based on cryptography. Quantum computers have close to unbounded computing capabilities and can easily break encryption and decryption keys. The ability to guess the secret keys or perform a quick reverse calculation using a quantum computer enables breakage of such keys, and this gives unauthorized or disguised network users the ability to intercept ongoing data transmissions or access the data [4]. With that being said, it is worth noting that quantum computing is limited to some extent as it cannot break all kinds of cryptographic algorithms. This means that it is one of the technologies which jeopardizes some of existing systems based on cryptography. Furthermore, the processes involved in cryptography can impose delays which can be unwelcome in some applications, such as fifth-generation (5G) ultra-reliable low-latency communication (URLLC) [24]. Moreover, cryptographic methods are inefficient in terms of energy consumption as they require extra resources for performing computations. Therefore, this calls for the need to implement new measures with an effort to augment cryptography. One of the technologies directed towards augmenting cryptography is physical layer security (PLS) [6,7,25].

**Figure 2:**
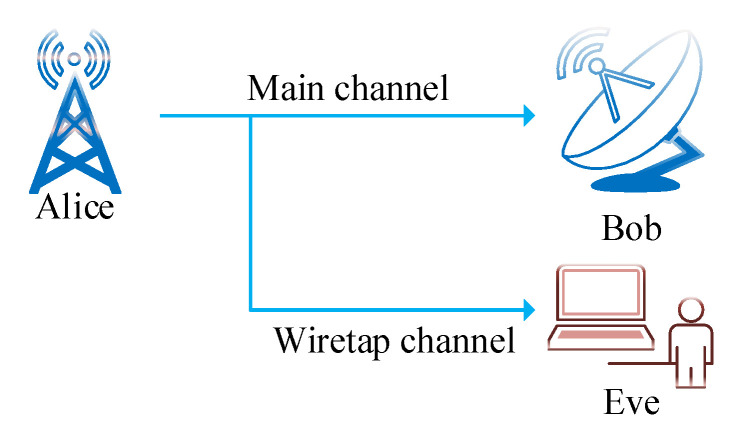
Wiretap channel.

**Figure 3:**
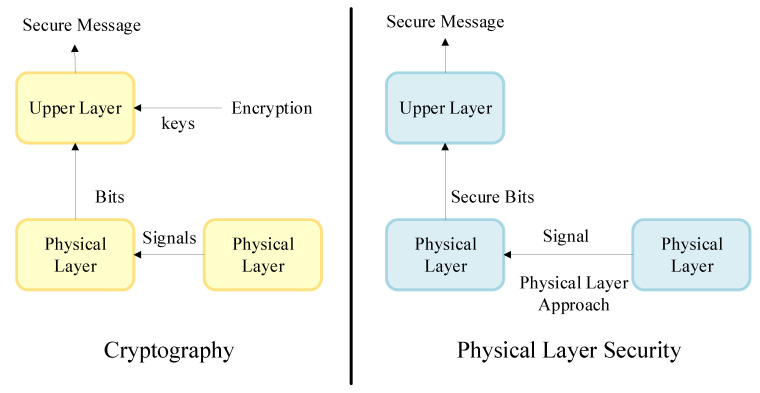
The difference between cryptography and physical layer security approaches [28].

PLS is different from cryptography technology because it is based on the concept of information-theoretic security proposed by Wyner [26]. The concept of PLS describes communication between two authorized users in the presence of an unintended user by modeling a discrete memory-less wiretap channel [27]. Figure 2 shows the general case of the wiretap channel where two authorized users communicate over the main channel and are observed by an eavesdropper through a wiretap channel. Figure 3 illustrates the fundamental differences between cryptography and PLS [28].

In contrast to cryptography, PLS schemes can seamlessly prevent unintended users from intercepting data signals. PLS is able to facilitate security without any form of encryption in the upper layers. The facilitation of key-free encryption is made possible by the exploitation of some wireless channel characteristics through the application of suitable signaling and channel coding [29]. PLS techniques have proven capable of realizing verifiable security even when the network intruders have almost limitless computational resources. Despite the unparalleled benefits of PLS, it is worth noting that some shortfalls exist. It was shown in [30] that it is almost impossible to warrant maximal security with a probability of one since PLS relies mainly on the average information. In addition, most PLS schemes assume prior knowledge of the eavesdropper’s wiretap channel, which is not feasible in practical applications. Furthermore, it is also worth noting that it will be difficult to only use PLS in future wireless systems since it requires a high data rate to ensure security. PLS can be combined with other higher-layer security techniques to achieve security and robustness of wireless communication networks. Authors in [31], proposed cross-layer cooperation as a viable solution for the achievement of reliability and energy efficiency in wireless communication. Chen et al. in [32] also investigated a cross-layer optimization scheme using cooperative diversity for reliable data transfer in wireless sensor networks to achieve significant energy savings and prolong the network lifetime considerably. Having stated the aforementioned benefits of both technologies, investigations on the concurrent use of PLS and cryptography are worth looking into to provide elevated robustness of the communication network.

## 2. Background

### 2.1. Concept and Evolution of PLS

**Figure 4:**
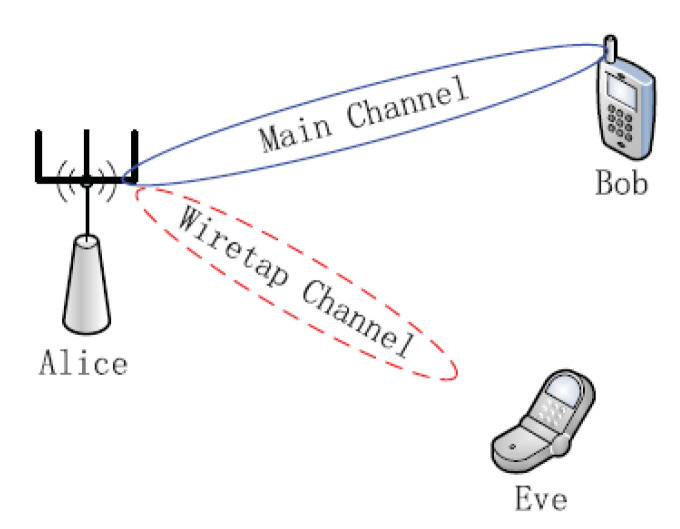
Physical layer security (PLS) system model.

PLS dates back to the 1970s with a mathematical description of a wiretap channel [26]. Following the advancements in massive MIMO and integration of technologies such as IEEE 802.11n and long-term evolution (LTE), there has been major interest in PLS research over the last decade. The current research is focused towards exploiting MIMO spatial degrees of freedom in order to leverage security benefits [33]. A typical network in which PLS is employed comprises three nodes: a transmitter, a legitimate receiver, and an eavesdropper. With this setup, the transmitter under normal circumstances sends a confidential message to the receiver. The sent signal is protected from any form of interception by the eavesdropper [29]. The adopted general convention is that the transmitter is referred to as “Alice”, whilst the eavesdropper and the receiver are referred to as “Eve” and “Bob”, respectively. This model is illustrated in Figure 4.

### 2.2. Motivation of PLS

It was shown in Section 1 that the inception of technologies such as quantum computing poses a major threat in existing security techniques in wireless networks, which means that wireless communication is not completely secure. PLS offers an additional solution to exploit the secrecy possibilities that the wireless channel offers. The main motivations behind PLS are (1) to find an alternative means to supplement the existing security measures in wireless networks which are based on cryptography algorithms, (2) employment of the physical layer of the network to improve security through enhancements of security methods implemented at the upper layer of the protocol stack, and (3) to find and develop new security technologies compatible with recent developments of the 5G and 6G wireless networks as well as MIMO communication.

### 2.3. Information-Theoretic Security

This is the kind of security that purely defines the fundamental limits of PLS measures from an information theory point of view. It was proposed by Claude Shannon in 1949 [34]. Shannon defined the channel capacity as the maximum rate at which information can be transmitted over a communication channel with an arbitrarily low probability of error. For an additive white Gaussian noise (AWGN) channel, the channel capacity is given by

(1) $$C=Blog_{2}\left(1+\mathrm{SNR}\right)$$

where *B* represents the channel’s bandwidth (fixed quantity) in Hertz (Hz), and SNR is the signal-to-noise ratio. The SNR is mathematically denoted by Equation (2). From Equation (1), it can be seen that the channel capacity is directly proportional to the power of the signal.

(2) $$\mathrm{SNR}=\frac{P}{\sigma^{2}},$$

where *P* denotes the power of the signal and $\sigma^{2}$ is the noise power.

**Figure 5:**
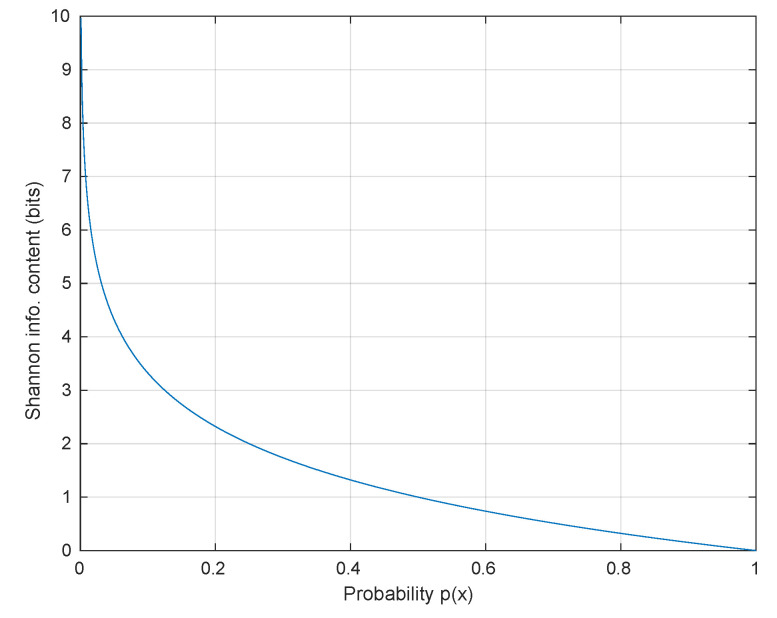
Shannon information content.

The Shannon information content of an outcome, $x_{i}$, is defined as

(3) $$h\left(x_{i}\right)=\log_{2}\left(\frac{1}{p(x_{i})}\right)=-\log_{2}p\left(x_{i}\right)$$

where the probability of the random variable *X* is denoted by $p(x_{i})$. Figure 5 shows a graph of Shannon information content versus the different probabilities, and it illustrates that less probable or rare outcomes contain more information than common or highly probable outcomes. The entropy function H(X) is the average of the Shannon information content and is given by

(4) $$H\left(X\right)=\sum_{i=1}^{M}p\left(x_{i}\right)\log_{2}\left(\frac{1}{p(x_{i})}\right)=-\sum_{i=1}^{M}p\left(x_{i}\right)\log_{2}p\left(x_{i}\right)$$

where *M* is the total number of possible outcomes. The mutual information, I(X;Y), defines the amount of information *X* conveys about *Y*:

(5) I(X;Y)=H(X)−H(X∣Y)

Therefore, this implies the amount of information sent to Bob by Alice is given by Equation (6), whereas the same quantity between Alice and Eve is given by Equation (7).

(6) I(A;B)=H(A)−H(A∣B),

(7) I(A;E)=H(A)−H(A∣E),

where random variable *A* represents the bit sent by Alice. *B* denotes the bits received by Bob, and the outcome observed by Eve is denoted by *E*. In order to ensure that the communication channel is secure, it is significant to maximize the achievable mutual information by optimizing the input distribution $p_{\left(A\right)}$ such that [35]

(8) $$C_{S}=\max_{p_{\left(A\right)}}\left(I\left(A;B\right)-I\left(A;E\right)\right)$$

Therefore the difference between the capacities of Bob and Eve’s channels gives the secrecy rate of the PLS system model given in Figure 4 by

(9) $$R_{s}=C_{B}-C_{E}$$

**Figure 6:**
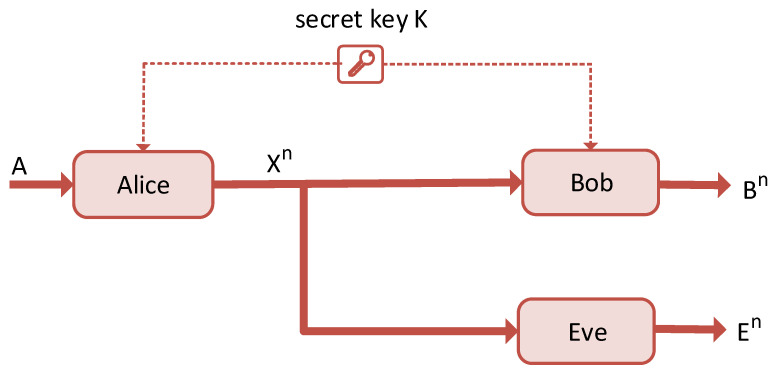
Shannon’s wiretap channel.

The notion of secrecy capacity plays a central role in PLS. It is a metric that defines the rate at which the transmitted signal reaches the legitimate receiver without any form of invasion from the eavesdropper. To define perfect secrecy, we consider Shannon’s wiretap channel model shown in Figure 6. In this model, Alice intends to transmit confidential information, *A*, to an authentic receiver, Bob, under a condition that an eavesdropper exists, Eve. *A* is encoded into $X^{n}$, which represents the information vector of length *n*. The received information vectors for Bob and Eve are given by $B^{n}$ and $E^{n}$, respectively. Consequently, the source information entropy together with the amount of uncertainty of the message received by Eve are given by H(A) and H(A∣$E^{n})$, respectively. Shannon showed that legitimate parties could achieve information-theoretically secure communication in a wireless communication environment by using the same random secret key, which is unknown to the eavesdropper. This is known as perfect secrecy, and it is given by Equation (10) in terms of the entropy. Transforming Equation (10) into Equation (11) shows that the eavesdropper is not able to receive any of the transmitted information content.

(10) $$H\left(A|E^{n}\right)=H\left(A\right)$$

(11) $$I(A,E^{n})=0$$

The theorem also proves that perfect secrecy could be guaranteed if H(K)≥H(A), where *K* is the random variable modeling the key. This means that the key should be equal to or longer than the confidential message [36]. However, perfect secrecy was proven to be impractical because the key management may be cumbersome for specific networks, such as ad hoc networks, which do not operate under fixed infrastructures [37].

**Figure 7:**
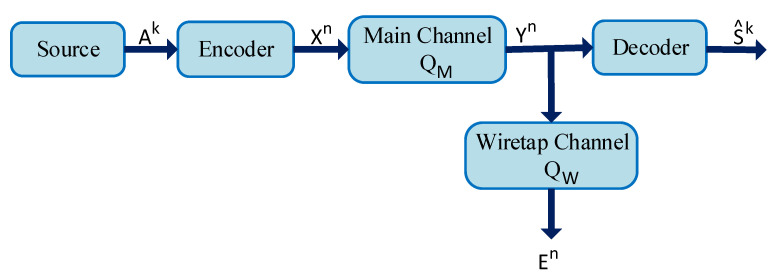
Wyner’s wiretap channel.

To expand Shannon’s information-theoretic secrecy theorem, Wyner proposed the concept of weak secrecy. Weak secrecy is defined by the Wyner’s wiretap channel model shown in Figure 7. In this model, the encoder operates on blocks of *k* source bits $A^{k}=\left(A^{1},A^{2},\ldots ,A^{k}\right)$ and produces an encoded sequence $X^{n}=\left(X^{1},X^{2},\ldots ,X^{n}\right)$ of length *n*.

The transmission rate, which is the proportion of information sent in each codeword, is given by Equation (12). The equivocation rate, defined as a measure of confusion at the eavesdropper, is employed to investigate the weak secrecy of transmitted information. The said rate is given by Equation (13).

(12) R=k/n(bits/channel),

(13) $$\Delta =\frac{1}{k}H\left(A^{k}|E^{n}\right),$$

where *k* is the total number of source bits in the code, and *n* is the code length. A scheme is considered to have weak secrecy in the event that

(14) $$\lim_{n\to \infty }\frac{1}{n}I\left(A,E^{n}\right)=0$$

**Figure 8:**
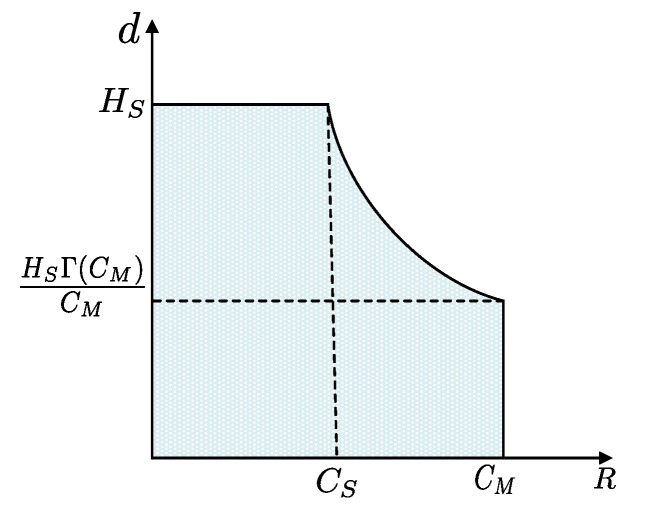
Wyner’s transmission rate vs equivocation rate.

Unlike the Shannon perfect secrecy metric, this metric proved that it is possible to obtain secrecy in a practical scenario [38]. Figure 8 illustrates the relationship between the transmission rate and equivocation. It shows the region of achievable pairs as (R,Δ) [26]. The highest rate capable of achieving complete equivocation $H_{s}$ (i.e., confusion at the eavesdropper) is the secrecy capacity $C_{s}$ of the channel.

With Wyner’s model, the assumption is that the signal arriving at the receiver of the eavesdropper is degraded by some degree in comparison to that of the legitimate receiver [36]. However, it was proved in [39] that secret communication is possible regardless of the statistical channel state of the eavesdropper. It was further shown in [30] that weak secrecy was also insufficient in many cases to prove secrecy in communication. Another metric, referred to as strong secrecy, was defined by [40]. The metric states that a scheme is said to achieve strong secrecy if

(15) $$\lim_{n\to \infty }I\left(A,Z^{n}\right)=0$$

Subsequently, in [41], strong secrecy was proved to be inefficient for some applications. This is based on the assumption of random and uniformly distributed message symbols over the message alphabet at the input of the secrecy encoders. In practice, the limitation is caused by the unavailability of universal compression algorithms capable of providing messages that have the distribution mentioned above.

### 2.4. Performance Metrics in PLS

The secrecy metrics from information-theoretic security are well-established and have laid the foundation of secrecy coding in the physical layer. However, they are challenging to evaluate and measure, especially when the coded sequence has a finite block length [42]. Therefore, different metrics that are much easier to work with have been proposed. In this subsection, we discuss some of these metrics according to how they are used to evaluate performance in PLS.

#### 2.4.1. Secrecy Rate

The rate of transmission that can reliably be supported on the legitimate channel of transmission but not decodable on the channel of the eavesdropper is termed secrecy rate. The secrecy rate of the Gaussian channel is calculated as the maximum difference between the attainable secrecy rates of Alice–Bob and Alice–Eve. Considering Figure 4, and using Equations (1) and (9), the secrecy rate of a typical PLS communication system is given by

(16) $$R_{s}=R_{B}-R_{E}=\log_{2}\left(1+\frac{P_{T}H_{B}}{\sigma_{B}^{2}}\right)-\log_{2}\left(1+\frac{P_{T}H_{E}}{\sigma_{E}^{2}}\right),$$

where $R_{B}$ and $R_{E}$ are the secrecy rates of Bob and Eve, respectively. The secrecy rate, $R_{s}$, can be maximized using signal design and optimization techniques, which will be discussed in Section 3.

#### 2.4.2. Secrecy Outage Probability (SOP)

SOP defines the probability at which a specific value of $R_{s}$ for a particular system is not obtainable. This metric is used in instances where Alice has very little channel state information (CSI) of Bob and Eve. In most of the applications, the SOP is put to use under the conditions where the eavesdropper’s statistical CSI is known to the transmitter. Additionally, the metric characterizes the reliability and the security of data transmission.

#### 2.4.3. Quality of Service (QoS)-Related Metrics

Signal-to-interference-plus-noise ratio (SINR): This metric may be described as the quantitative relationship between the power of the received signal and power of the interference plus noise. The performance of a communication link is characterized by the QoS. SINR is directly related to the QoS, and this can aid with the design of secrecy algorithms. A minimum value of SINR for transmission from Alice to Bob and a maximum value of SINR for Eve means a good receiving performance in terms of security and reliability. This paves the way for a robust transmission capable of achieving the desired minimum and maximum error levels for Bob and Eve, respectively. Improvement of the SINR can facilitate the use of PLS techniques such as beamforming. A detailed discussion of beamforming is given in Section 3.

Bit error rate (BER): This metric is defined as the ratio of the number of information bits received in error to the total number of bits transmitted. Different modulation and coding techniques have varying BER performances at the same SINR level. For the establishment of a communication link, the BER of a system must be above the minimum required level. Therefore, PLS techniques utilize optimization methods to improve the security of a communication system by degrading the BER of illegitimate users. Thus, the BER can be employed to measure the QoS and the security of a communication system.

### 2.5. Channel State Information (CSI)

CSI defines the properties of a channel in a wireless communication link. CSI is used to describe the propagation of the transmitted signal in relation to the corresponding effects such as scattering, fading, and power decay with distance. Wang et al. in [29] show that the availability of CSI is one of the most important aspects to consider when choosing an appropriate secrecy performance metric in order to design optimal transmission strategies in PLS. CSI can be categorized into two classes, and they are referred to as perfect and imperfect CSI. The former involves the complete knowledge of the channel properties of a communication link. The latter is concerned with characterization of the statistical information only. Such information includes the average channel gain, the type of fading distribution, the line-of-sight (LOS) component, and the spatial correlation.

In practice, CSI is initially not available at the transmitter and receiver terminals; it is gathered through some channel estimation methods, such as those found in [43,44]. The two channel estimation mechanisms commonly employed to acquire CSI are the pilot-based channel training and channel state feedback. In pilot-based channel training, the transmitter distributes the total transmission time and energy such that some of the energy and time are allocated for the transmission of pilot symbols while the remaining portion is used for data transmission. In the channel state feedback method, the receiver is required to share its estimated channel knowledge with the transmitter prior to transmission. It is shown in [42] that the greatest level of security can be achieved if the transmitter has full knowledge of both the wireless channels to the intended user and the unintended user. This can be achieved by designing MIMO transmit precoders that minimize the information leaked to the eavesdropper’s channels or to accurately direct jamming signals towards the eavesdroppers. A detailed discussion on the use of CSI in PLS is given in Section 3.

### 2.6. Multiple-Input Multiple-Output (MIMO) Communication

**Figure 9:**
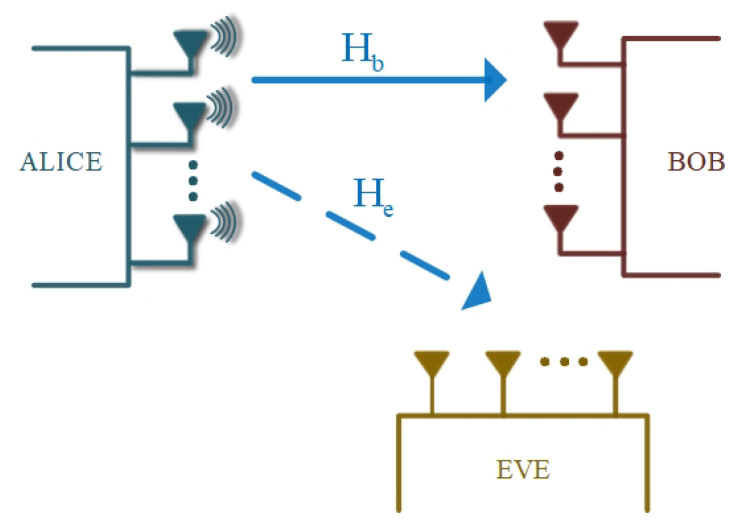
MIMO wiretap channel model [29].

MIMO is a very powerful technology in wireless communication systems. A MIMO network structure consists of many antennas at the transmitter as well as at the receiver. MIMO technology has been well-studied and developed for the past decade, mainly because of its capability to significantly enhance performance and widen the coverage range of wireless communication systems [45]. Some of its other important benefits that have been shown include, but are not limited to, its capability to provide higher data rates, improved reliability, and less noise and interference [46]. Even though MIMO technology was proposed many years back, it only came to reality in the practical world in 2018 [47]. In this review paper, we focus on the relevance and application of MIMO technology in PLS rather than its traditional capacity benefits in wireless communication networks. It was shown in [48] that MIMO systems are very robust against passive eavesdropping attacks since the secrecy capacity is directly proportional to the difference in capacities of the main and wiretap channels. Beamforming in massive MIMO was used in [49] to implement security in the physical layer. In [50], artificial noise generation was used to demonstrate the application of PLS in MIMO orthogonal frequency division multiplexing (OFDM) systems. A typical MIMO system is shown in Figure 9 wherein the transmitter, the intended receiver, and the eavesdropper are equipped with multiple antennas $N_{A}$, $N_{B}$, and $N_{E}$. This is referred to as MIMO multiple-antenna eavesdropper (MIMOME) [29], and in this case the secrecy capacity can be expressed as

(17) $$C_{s}=\max_{K_{x}≻0,tr\left(K_{x}⪯P\right)}\frac{\log_{2}|I+H_{b}K_{x}H_{b}^{H}|}{\log_{2}|I+H_{e}K_{x}H_{e}^{H}|}$$

where I is the identity matrix, $K_{x}$ is the covariance matrix of the transmit signal *x*, *P* is the maximum transmit power constraint, and $H_{b}\in C^{N_{B}\times N_{A}}$ and $H_{e}\in C^{N_{E}\times N_{A}}$ are the MIMO complex Gaussian channel matrices of the legitimate and wiretap channel, respectively. The signals received by the legitimate receiver and passive eavesdropper are given by Equation (18):

(18) $$\begin{array}{cc} \; & y_{b}=H_{b}x_{a}+n_{b},\\ \; & y_{e}=H_{e}x_{a}+n_{e}, \end{array}$$

where $x_{a}\in C^{N_{A}\times 1}$ is the transmit signal, and $n_{b}\in C^{N_{B}\times 1}$ and $n_{e}\in C^{N_{E}\times 1}$ are zero-mean complex white Gaussian additive noise vectors. Equation (18) is used as a fundamental tool in optimization techniques in PLS and also to demonstrate the application of MIMO in Section 3.

## 3. Secure Multi-Antenna Techniques

**Figure 10:**
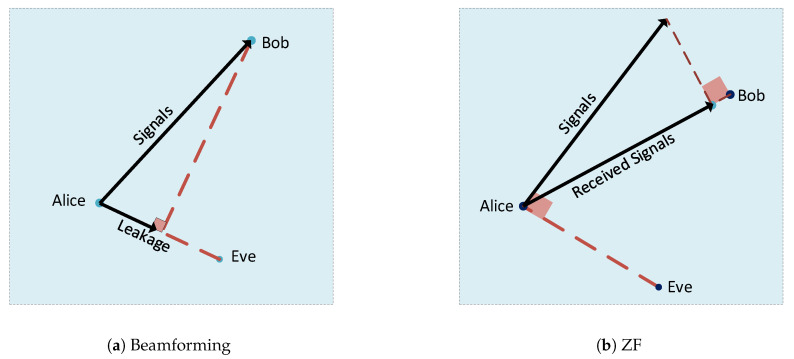

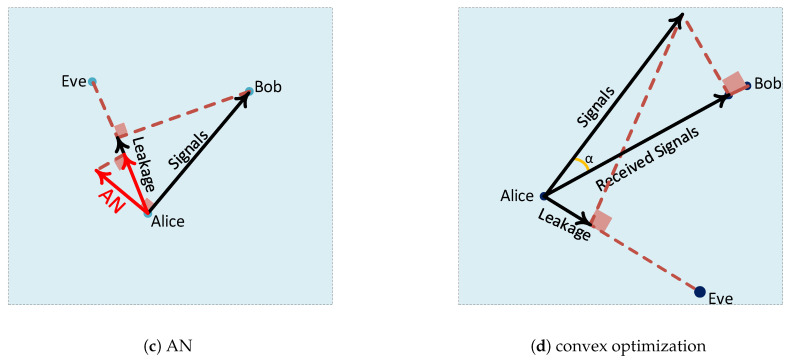
Secure multi-antenna techniques.

Multi-antenna techniques have been widely considered in wireless communication because they offer higher spatial degrees of freedom, which can be utilized effectively in PLS to ensure secure data transmission. Such techniques either attempt to degrade the eavesdropper’s channel relative to the main transmission channel or enhance the quality of the received signal at the legitimate receiver. From the perspective of optimization, the four techniques which are representative of this area are (1) beamforming, (2) zero-forcing (ZF), (3) convex optimization, and (4) artificial noise (AN). In Figure 10, from [33], each technique is described in terms of the transmission’s orthogonality to Bob and Eve.

### 3.1. Convex Optimization

Convex optimization can be used in PLS with other secure-multi antenna techniques to find the most favorable transmit solutions that can effectively make the best out of the performance metrics of the wireless communication system. The objective function f(x) to be maximized or minimized may be considered to be the performance metric, for example, secrecy rate, SINR, and secrecy outage probability [7]. Several methods which are commonly used to solve optimization problems in PLS are widely available in the literature [51].

One convex optimization method involves the objective function having quadratic terms, and it is called quadratic programming. Quadratic programming is used in the design problems of nonlinear programming. Some of the common quadratic problems in PLS are power minimization, secure power allocation, and beamforming. Another method used in PLS is semi-definite programming (SDP). It is used to optimize a linear function of variables under linear equality constraints and a non-negativity constraint. Most problems in PLS are usually non-convex, and they must be converted into convex problems using SDP. In turn, an efficient algorithm that is easy to implement is developed in order to obtain optimal performance metrics. One other convex optimization method is the difference of convex functions (DC) programming. In DC programming, the objective function is a subtraction of two convex functions, for instance, secrecy rate maximization. Furthermore, mixed-integer programming is one of the methods utilized. This method is applicable to problems which have discrete and continuous variables. Not least of all, fractional programming is one more method which is directed towards optimization of a ratio of two nonlinear functions. A typical example of its application in PLS is the energy efficiency maximization. Even though convex optimization offers improved secrecy performance in the physical layer than the conventional precoding techniques such as beamforming or zero-forcing, it is more computationally expensive to implement.

### 3.2. Beamforming

**Figure 11:**
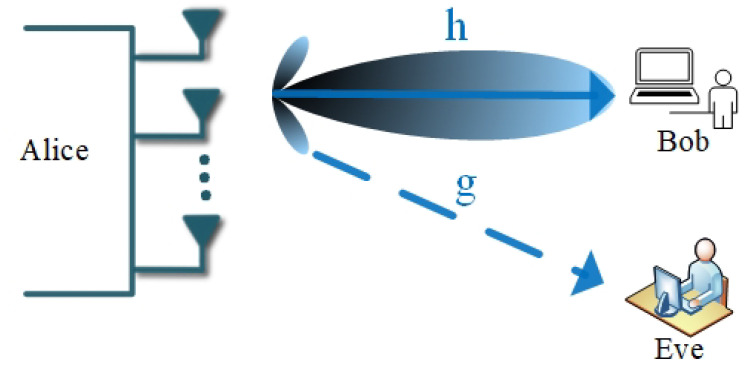
Beamforming.

Beamforming is a signal processing technique that is used to transmit signals effectively in intended directions to give a maximum signal difference between the receiver in the intended direction and the one in the unintended direction. Beamforming forms a beam in the direction of the desired recipient to maximize the signal-to-noise power ratio while suppressing the reception or transmission in the direction of the unintended user, Figure 11. This significantly improves the energy efficiency of the system because the energy is transmitted or focused in a particular direction rather than being spread out in a diffused fashion.

Beamforming can be used at both the transmitting and receiving ends to achieve spatial selectivity, i.e., transmit beamforming and receive beamforming. Transmit beamforming steers the transmitted signal towards the intended receiver by finding the best possible channel among all the transmit antennas.

**Figure 12:**
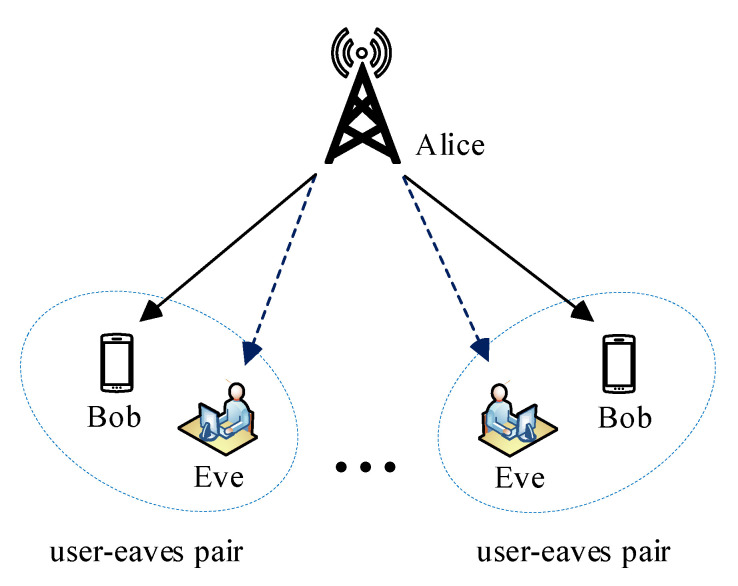
System model of multi-user and eavesdropper pairs with beamforming.

Beamforming is one of the key techniques in PLS and has been widely studied in the literature [49,52,53,54,55,56,57]. A beamforming problem in PLS involves steering the transmitted signal towards the desired user while taking into account an interfering user trying to decode the transmitted information, Figure 11. To demonstrate the beamforming optimization problem in PLS we consider a MISO system shown in Figure 12 in which the transmitter uses transmit beamforming to communicate with *K* users.

We assume that the transmitter is equipped with $N_{t}$ transmit antennas, and the legitimate user and the eavesdropper each have a single receiving antenna. Therefore, the received signal at legitimate user *i* and their equivalent SINR are given by

(19) $$\begin{array}{cc} \; & y_{i}=h_{i}^{H}x_{i}+\sum_{\begin{array}{c} k=1\\ k\neq i \end{array}}^{K}h_{i}^{H}x_{k}+n_{i},\\ \; & \mathrm{SINR}=\frac{{\left|h_{i}^{H}w_{i}\right|}^{2}}{{\left|\sum_{\begin{array}{c} k=1\\ k\neq i \end{array}}^{K}h_{i}^{H}w_{k}\right|}^{2}+\sigma^{2}} \end{array}$$

Similarly, the signal received signal by the $i^{th}$ eavesdropper and their equivalent SINR can be given by

(20) $$\begin{array}{cc} \; & z_{i}=g_{i}^{H}x_{i}+\sum_{\begin{array}{c} k=1\\ k\neq i \end{array}}^{K}g_{i}^{H}x_{k}+m_{i},\\ \; & \mathrm{SINR}=\frac{{\left|g_{i}^{H}w_{i}\right|}^{2}}{{\left|\sum_{\begin{array}{c} k=1\\ k\neq i \end{array}}^{K}g_{i}^{H}w_{k}\right|}^{2}+\sigma^{2}} \end{array}$$

where $x_{i}\in C^{N_{T}\times 1}$ is the transmitted signal symbol of the desired user *i* with corresponding beamforming vector $w_{i}\in C^{N_{T}\times 1}$, $h\in C^{N_{T}\times 1}$ and $g\in C^{N_{T}\times 1}$ are the channel vectors of the desired user and eavesdropper respectively, and $n_{i}$ and $m_{i}$ are the corresponding AWG noise vectors for the $i^{th}$ user and eavesdropper with zero mean and noise power $\sigma^{2}$. The covariance matrix of the transmitted signal is given by $R_{n}=E\left\{x_{i}x_{i}^{H}\right\}$. The objective problem of a typical beamforming design scheme is to minimize the interference signal at the desired user so that they receive the transmitted signal with the desired QoS, which is usually described by the constraint of the SINR greater or equal to the given threshold amount of the $i^{th}$ user, i.e., $\mathrm{SINR}\geq \rho_{i}$. Therefore, the beamforming optimization problem can be written as follows:

(21) $$\begin{array}{cc} \min_{w_{i}}\; & {\left|\sum_{\begin{array}{c} k=1\\ k\neq i \end{array}}^{K}h_{i}^{H}w_{k}\right|}^{2}+\sigma^{2}\\ s.t.\; & \mathrm{SINR}\geq \rho_{i}\\ \; & {\left|\left|w_{i}\right|\right|}^{2}\leq P_{T} \end{array}$$

The solution to the beamforming problem provides the optimal vector $w_{i}$, which maximizes the SNR of the desired user. This results in focusing the beam in one direction, and the process is referred to as electronic steering. One beamforming vector is assigned to each legitimate user and is matched to their channel. The beamforming design problem in PLS has been well-investigated in many studies in the literature, which can be found in [51,58,59,60,61,62,63,64,64], with the aim of developing algorithms that minimize the interference and also maximize secrecy of transmission. We summarize some of the results from these studies here. One approach which was considered in [58] involves using semi-definite relaxation to obtain the optimal beamforming solution, which minimizes the transmission power subject to SINR constraints. In the paper, they showed that the quadratic optimization problems with non-convex and discontinuous constraints could be recast as SDP with additional constraints, which imposes that the solution matrices must be of rank one. Another study employing semi-definite relaxation was proposed in [59], wherein they used Taylor expansion to solve the optimization problem. The author’s study proved that their proposed algorithm outperformed both the signal-to-leakage-and-noise ratio (SLNR)-based algorithm and zero-forcing beamforming. The SLNR algorithm and zero-forcing were employed to minimize the power leaking to the channels of other users. Another novel approach of path-following algorithm was proposed in [60,61], which used a simple quadratic program to perform iterations for finding the optimal transmit beamformers. The QoS, which is given in terms of both the user’s secrecy throughput and the network secure energy efficiency, is optimized through the use of the obtained beamformers. The algorithm proposed in [60,61] offers a better performance when compared with the existing methods based on zero-forcing beamformers. Authors in [51,51] further studied the algorithm proposed by [60,61] using a different approach. The authors defined secrecy throughput in terms of outage probability. It was found that this approach offered a more practical beamforming design solution. Interference alignment (IA) was proposed by [65,66] as an excellent solution for interference management in multi-user wireless networks to significantly improve the sum-rate. The concept of the IA technique is that the transmitted signals are directed to concentrate the interference in the particular sub-spaces at the unintended receivers, thus opening up interference-free sub-spaces to transmit the desired signal to the intended user. Following the proposal, several research works in [64,67,68,69] have been conducted to develop IA algorithms for directing the interference in a manner that is detrimental to the illegitimate receiver while ensuring that the legitimate receiver is not severely affected. To enhance the desired signal gain and suppress the undesired interference and the noise signal, authors in [63] employed spatial degrees of freedom.

### 3.3. Artificial Noise (AN) Precoding

**Figure 13:**
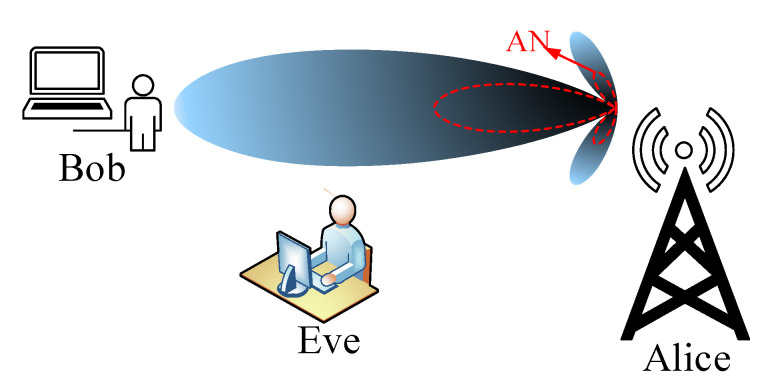
Secrecy beamforming with artificial noise (AN).

The notion of using artificial noise to enhance security in the physical layer was first proposed in [70]. They identified AN-based transmission as an effective technique that can be deployed in PLS to ensure secure communication in wireless networks. The technique involves deliberately degrading the quality of the channel of the eavesdropper by generating an interference signal, which is used to interrupt their eavesdropping capabilities. In the AN precoding scheme, the transmitter Alice divides the transmission power between transmitting the information to the intended recipient, Bob, and transmitting the noise signal towards the eavesdropper, Eve, is shown in Figure 13.

Generating AN depends on the transmitter’s knowledge of the eavesdroppers’ channel state information. In a case where the eavesdropper’s CSI is unknown, the isotropic AN is generated. The generated AN is designed such that it lies in the nullspace of the intended receiver and directed in the range space of the unintended receivers. This is done to cancel out its effect at the intended receiver such that only the eavesdropper’s channel is degraded [70]. Another form of AN generation is called spatially selective AN, which is applicable in the event that the transmitter knows the the eavesdropper’s CSI [71]. The AN generation technique’s major strength is that the provided secrecy scales well with the SNR since an increase in SNR at Eve will increase the received AN power along with the message power. The conventional AN scheme can be represented in general as follows:

(22) $$x=ws_{a}+vs_{j},$$

where x is the signal transmitted by Alice. The source information is denoted by $s_{a}$ and $s_{j}$ denotes the AN jamming signal, which is chosen to be independent of the source information, i.e., $s_{a}\neq s_{j}$. Beamforming vectors for the information and jamming signals are represented by w and v, respectively. Therefore, the signals received by Bob and Eve are given by Equations (23) and (24), respectively.

(23) $$y_{B}=h_{B}^{H}ws_{a}+g_{B}^{H}vs_{j}+n_{B}$$

(24) $$y_{E}=h_{E}^{H}ws_{a}+g_{E}^{H}vs_{j}+n_{E}$$

where $h_{B}^{H}$ and $g_{E}^{H}$ denote the channel responses of Alice–Bob and Alice–Eve, respectively. The independent, identically distributed complex Gaussian noise for Bob and Eve with zero mean and variance $\sigma^{2}$ are denoted by $n_{B}$ and $n_{E}$, respectively. The corresponding secrecy rate is given by

(25) $$R=\log_{2}\left(1+\frac{{\left|h_{B}^{H}w\right|}^{2}}{{\left|g_{B}^{H}v\right|}^{2}+\sigma^{2}}\right)-\log_{2}\left(1+\frac{{\left|h_{E}^{H}v\right|}^{2}}{{\left|g_{E}^{H}v\right|}^{2}+\sigma^{2}}\right)$$

A novel approach of the AN scheme that offers better secrecy rate performance, where the jamming signal is generated to be dependent on the information signal, was presented in [72]. The scheme significantly improved the signal strength at Bob and, at the same time, canceled the received signal at the eavesdropper. In the scheme the signal received by Bob and Eve can be shown by

(26) $$y_{B}=h_{B}^{H}ws_{a}+g_{B}^{H}vs_{a}+n_{B}$$

(27) $$y_{E}=h_{E}^{H}ws_{a}+g_{E}^{H}vs_{a}+n_{E}$$

Therefore, the secrecy rate is now given by

(28) $$R=\log_{2}\left(1+\frac{{\left|h_{B}^{H}w+g_{B}^{H}v\right|}^{2}}{\sigma^{2}}\right)-\log_{2}\left(1+\frac{{\left|h_{E}^{H}v+g_{E}^{H}v\right|}^{2}}{\sigma^{2}}\right)$$

The optimization problem in the AN precoding scheme is to find an optimal power allocation method for the artificial noise, which ensures maximum secrecy of the legitimate transmission. A comprehensive summary of several methods proposed in the literature for solving the AN precoding optimization is presented below.

The classical AN injection schemes are investigated broadly in the literature, and their application in MIMO systems has been shown as promising to exploit in future wireless networks. In [73], the authors demonstrated an approach capable of guaranteeing secrecy without knowledge of the eavesdropper’s CSI. In [73], two schemes were proposed for AN generation in PLS. In the first scheme, they proposed a scheme which was based on MIMO technology, while the second scheme used a single transmitter antenna. For the latter scheme, amplifying relays were used to mimic the effects of multiple antennas. Moreover, in the second scheme the transmitter and the intended receiver both transmit independent AN signals to the helper nodes. The eavesdropper receives differently weighted versions of the AN signals from the transmitter and the receiver. The two transmission schemes proposed in the paper transmit both AN and information-bearing signals together. However, the paper does not necessarily find an optimal power allocation for the transmission of message signals and minimal power allocated to the AN.

To tackle the shortfall indicated in [73], researchers [74] proposed an AN-assisted secure MIMO-OFDM system to improve the security of the legitimate transmission and find an optimal power allocation scheme. AN precoding scheme is proposed where Alice divides her power between transmitting a message to Bob and transmitting AN into Bob’s nullspace. Assuming Bob and Eve’s channels are independently faded, Eve will see some of the AN in her range space. The authors determined the minimum power consumption that satisfies the legitimate transmission quality with the largest residual power in generating AN. They use convex optimization solvers to find the optimal solution to this problem. This technique’s major strength is that the provided secrecy scales well with SNR, since an increase in SNR at Eve will increase the received AN power along with the message power [23].

AN precoding was also used in [50] to implement security in the physical layer. AN was used as a transmit strategy over Bob’s null space with the intention of improving the secrecy of the Alice–Bob channel without affecting its quality. The authors examine three approaches of generating AN. Firstly, the minimum power was used for the information-bearing signal, and the rest of the transmit power was distributed to the AN. Secondly, the power was evenly distributed between the transmit signal and the AN signal in order to maximize ergodic secrecy capacity. Lastly, the AN power was progressively varied in order to understand its effects on the secrecy confidence level. Eve was modeled to use the minimum mean-square error (MMSE) as an optimal receiver structure to maximize the SNR. Eve’s capability to compromise the secrecy of the main link was defined as the SNR difference between the intended receiver and the eavesdropper. It was shown in [50] that when an eavesdropper uses zero-forcing to mitigate the interference introduced by the artificial noise transmission with a large number of receiving antennas and knows some of the main channel’s CSI, the secrecy of the system can be compromised.

### 3.4. Zero-Forcing (ZF) Precoding

Zero-forcing precoding, or null-steering, is a method of spatial signal processing in which multiple antennas at the transmitter can completely cancel out or null the multiple user interference signals in a wireless communication network. The ZF method is based on canceling out the interference at the intended receiver in multiple user communications. This can be done by using the eavesdropper’s CSI to transmit a message orthogonal to the eavesdropper, which is equivalent to steering a null in the direction of the eavesdropper, illustrated in Figure 10b.

The design problem of ZF precoding has been presented in [75]. The iterative algorithm presented in [75] was developed to obtain the optimal transmit and receive filters to cancel out the interference. The designed filters minimizes the mean-square error (MSE) between the legitimate parties whilst guaranteeing and maintaining a certain eavesdropper MSE level, subject to the power constraint. Nonetheless, the ZF precoding methods presented have been demonstrated to perform well or achieve the highest secrecy system capacity when full knowledge of CSI is available at the transmitter, which is a highly unlikely case. Therefore, with limited CSI at the transmitter, the performance of ZF precoding is very poor, which makes it less applicable.

### 3.5. Cooperative Jamming

**Figure 14:**
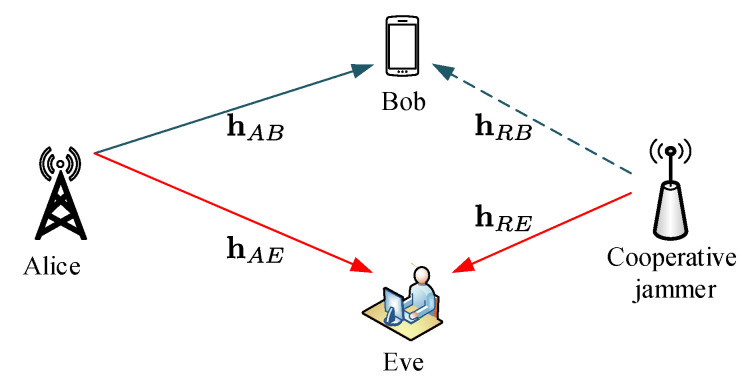
Secrecy with cooperative jamming.

Cooperative jamming is one of the techniques proposed to implement security in the physical layer to curb eavesdropping in wireless networks. A cooperative jamming network comprises the source that transmits its message to the intended receiver and a relay node that transmits a jamming signal to degrade the eavesdropper’s channel and improve the secrecy rate. Cooperative jamming was derived from the conventional technique for user cooperation known as cooperative relaying. However, cooperative relaying is distinctive from cooperative jamming because it improves the security by enhancing channel quality between the transmitter and legitimate receiver. Some of the examples of cooperative relaying are decode-and-forward (DF) and amplify-and-forward (AF) schemes. A system model of a typical cooperative jamming scheme is shown in Figure 14, which consists of the transmitter Alice, a single trusted relay, an intended receiver Bob, and and eavesdropper Eve. Bob transmits a message signal *s* using a transmit power PA, and the relay transmits a jamming signal *z* with a weighting vector w, simultaneously.

The signal received by Bob and Eve is given by Equations (29) and (30), respectively

(29) $$y_{B}=\sqrt{P_{A}}h_{AB}s+w^{H}h_{RB}z+n_{B}$$

(30) $$y_{E}=\sqrt{PA}h_{AE}s+w^{H}h_{RE}z+n_{E}$$

where $h_{AB}$ is the Alice–Bob channel, $h_{RB}$ is the relay–Bob channel, $h_{AB}$ is the Alice–Bob channel, $h_{AB}$ is the Alice–Bob channel, $h_{RE}$ is the relay–Eve channel, $h_{AE}$ is the Alice–Eve channel, and $n_{B}$ and $n_{E}$ are the AWG noise with variance $\sigma^{2}$ at both Bob and Eve, respectively. The corresponding secrecy rate is given by

(31) $$R=\log_{2}\left(1+\frac{P_{A}{\left|h_{AB}\right|}^{2}}{{\left|w^{H}h_{RB}\right|}^{2}+\sigma^{2}}\right)-\log_{2}\left(1+\frac{P_{A}{\left|h_{AE}\right|}^{2}}{{\left|w^{H}h_{RE}\right|}^{2}+\sigma^{2}}\right)$$

The problem of secrecy maximization has been extensively studied in the literature [76,77,78,79,80,81,82,83]. Authors in [76,77] investigated the problem of secrecy rate maximization of a secure wireless communication system in the presence of multiple eavesdroppers. Hu et al. in [79] studied cooperative jamming for PLS enhancement in IoT, specifically considering a downlink transmission problem to tackle multiple passive and non-colluding eavesdroppers. The current research in cooperative jamming is focused towards integrating cooperative jamming with the current technologies in 5G and 6G, which can be found in [80,81,82,83,84]. In [80], they used joint cooperative jamming and secure channel training solutions to safeguard a two-user power domain non-orthogonal multiple access (NOMA) system against eavesdropping attacks coming simultaneously from inside and outside of the network. Another interesting study in [81] uses cooperative jamming to implement security for industrial wireless networks with mobile users and eavesdroppers. In the paper they employed an edge computing device to intelligently select an optimal cooperative node.

### 3.6. Space-Time Coding (STC)

**Figure 15:**
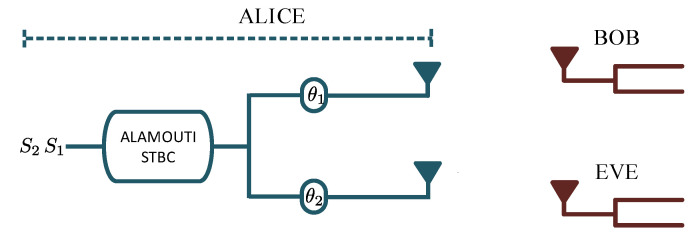
System block diagram.

In 1998, Alamouti proposed a space-time block code (STBC) to achieve transmitter diversity [85]. Space-time coding is based on the Alamouti scheme. In the conventional transmit schemes, diversity techniques were applied at the receiver using algorithms such as the MRC. These techniques require knowledge of the channel between the transmitter and the receiver to derive the optimal beamforming weights. However, Alamouti showed that it is possible to transmit data using multiple antennas and perform separation at the receiver using a single receiving antenna, which gives the same diversity gains. To provide diversity using the Alamouti scheme, the time and space blocks are used to encode the information signal. The Alamouti space-time encoder takes a block of two modulated symbols to create an encoding matrix denoted by

(32) $$C=\;\left[\begin{array}{cc} s_{1} & -s_{2}^{*}\\ s_{2} & -s_{1}^{*} \end{array}\right],$$

where $s_{1}$ and $s_{2}$ are the modulated symbols mapped to two transmit antennas in two transmit time slots [86]. The columns of C represent timeslots, and the rows represent different transmit antennas. Studies by [87,88] showed that STBC may be employed to provide secure communication for space-time systems while lowering the eavesdropper’s order of diversity. A technique depicted in Figure 15 to achieve a secure STBC without the need to estimate CSI at the transmitter was proposed in [87]. The proposed technique uses mutual received signal strength indicator measurements to generate a pseudo-random sequence used to secure communication. In this model, at each transmit antenna, random phase rotations $\theta_{1}$ and $\theta_{2}$ are applied to the symbols. Each phase shift is applied for one code duration. For a single codeword, the transmitter encodes source information $s_{1}$ and $s_{2}$ as

(33) $$X=\;\left[\begin{array}{cc} s_{1}e^{j\theta_{1}} & s_{2}^{*}e^{j\theta_{2}}\\ -s_{2}e^{j\theta_{1}} & s_{1}^{*}e^{j\theta_{2}} \end{array}\right]$$

Therefore, Bob receives the signal given by

(34) $$\begin{array}{cc} z & =Xh+n\\ z & =H^{+}\left(\theta_{1},\theta_{2}\right)s+\tilde{n}\\ \left[\begin{array}{c} z_{1}\\ -z_{2}^{*} \end{array}\right]\; & =\left[\begin{array}{cc} h_{1}e^{j\theta_{1}} & h_{2}^{*}e^{j\theta_{2}}\\ -h_{2}e^{j\theta_{1}} & h_{1}^{*}e^{j\theta_{2}} \end{array}\right]\left[\begin{array}{c} s_{1}\\ s_{2} \end{array}\right]+\left[\begin{array}{c} n_{1}\\ -n_{2}^{*} \end{array}\right] \end{array}$$

Using the MRC algorithm, the source information can be estimated by

(35) $$\tilde{s}=H^{+}\left(\theta_{1},\theta_{2}\right)z$$

Eve’s received signal is given by *y*, which can be also be decomposed to $\tilde{y}$ as

(36) $$\begin{array}{cc} y & =Xg+e\\ \tilde{y} & =G^{+}\left(\theta_{1},\theta_{2}\right)s+\tilde{e} \end{array}$$

where $H^{+}$ and $G^{+}$ are the pseudo-inverse of H and G, respectively. The transmitter manipulates the transmitted symbols by generating and applying the maximum number of phase rotations so that the eavesdropper is completely denied access to the source information. The strength of this technique is that improved security is achieved without the knowledge of the CSI, unlike in the preceding security schemes which assumes that the CSI is available. However, it was shown in [88] that the security of the transmitted signal is compromised if the eavesdropper can obtain one of the space angles and is in close proximity to Bob. To avoid this, the authors proposed a technique that was able to achieve zero diversity for the eavesdropper even under enhanced receive diversity. This was achieved through signal and space rotations.

**Figure 16:**
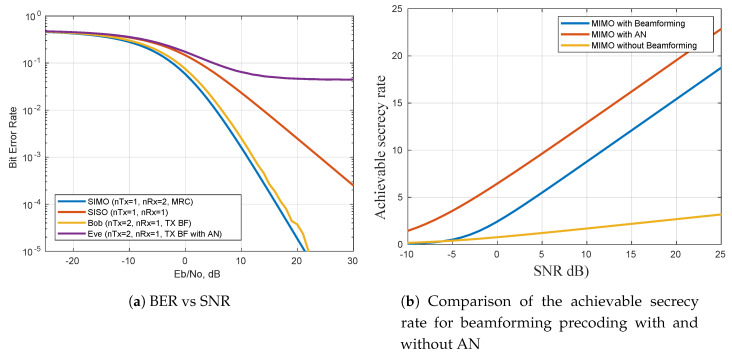
Bit error rate (BER) and secrecy rate performance metrics simulations for different precoding schemes.

In Figure 16a we demonstrate the BER performance of the beamforming and AN schemes. The figure illustrates that the eavesdropper’s channel has the worst bit error rate because it is corrupted by the AN signal; it is followed by a single-input single-output (SISO) system, which does not receive diversity from beamforming. Lastly, the 1×2 single-input multiple-output (SIMO) and 2×1 multiple-input single-output (MISO) systems receive the signal with better BER because of beamforming. The achievable secrecy rates of different precoding schemes are shown in Figure 16b. In particular, in a MIMO scheme, six transmit antennas, the intended receiver, and eavesdropper are each equipped with two receive antennas. In the figure we have the same MIMO configurations for no beamforming and use of beamforming. Lastly, we introduce an AN signal. The figure confirms that the use of AN indeed improved the secrecy rate of the communication network compared to beamforming only or no beamforming used.

## 4. Challenges and Promising PLS Solutions

### 4.1. Challenges and Limitations in PLS

The individual PLS techniques discussed above have their own different pros and cons. The major challenge is the ability to implement an optimal secure transmit precoding algorithm that can maximize the achievable secrecy rates without any counter cost. We outline some of the notable challenges in detail, and show how they are addressed, in the subsequent section.

One problem prevalent in PLS techniques, specifically beamforming, is the leakage of transmitted signals into the eavesdropper’s subspace. In beamforming, the transmitted signal is steered in the desired direction to the legitimate receiver. The transmit power is highly concentrated in the main lobe beam, but some of the power is lost in the minor side lobes. This leakage makes it possible for eavesdroppers who are in the vicinity to decode the transmitted signal since the finite number of transmit antennas could only provide a limited amount of spatial directivity [52]. It has also been shown that transmit beamforming focuses only on enhancing the quality of the main channel [89]. Beamforming does not take into consideration the possibility that the eavesdropper can have a favorable channel when compared to the main channel. Therefore, it can be concluded that even though the design of an optimal beamforming vector for the intended receiver is fairly easy, it is cumbersome and computationally expensive to come up with a perfect balance between getting rid of signal leakage and obtaining the optimal signal power.

It has been shown in Section 3.3 that AN precoding offers provable security in the physical layer. However, this comes at an additional cost of extra energy requirement. The approach relies on the generation of AN signals. A fraction of the power used for transmission of information signal power is used to generate the AN signal. This consumes the transmission power which could have been used to improve the channel capacity and receiver’s SNR. Therefore, there is a trade-off between capacity and secrecy rate capacity by means of the transmit power available. On the other hand, there is also a higher power consumption in both convex optimization and zero-forcing, owing to the computational requirements.

PLS precoding schemes make an assumption of the knowledge or availability of the unintended user’s CSI. This is the fundamental limitation of such PLS techniques. In practice it is very difficult for the transmitter to obtain the CSI of the eavesdropper. This is due to the fact that the eavesdropper does not naturally cooperate with the transmitter to send CSI feedback. Therefore, this assumption is entirely valid for theoretical systems. Most PLS secure precoding techniques assume that an eavesdropper has limited resources. To be more specific, the eavesdropper is commonly assumed to have a smaller number of antennas when compared to those of the intended receiver. Even though it has been proposed that secrecy against an eavesdropper with more antennas than the transmitter is possible in [90], such a solution requires additional power assumptions. Hence, secrecy against resourceful eavesdroppers remains a major challenge in PLS precoding.

### 4.2. Promising PLS Solutions

#### 4.2.1. Simultaneous Wireless Information and Power Transfer (SWIPT)

**Figure 17:**
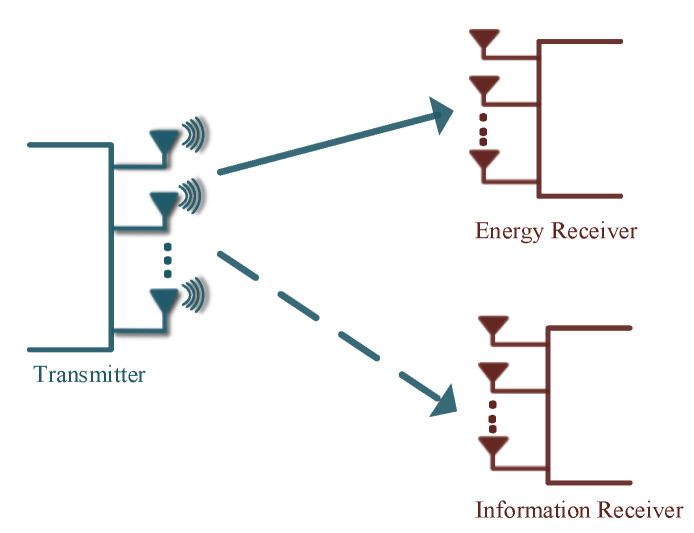
A multiple-input multiple-output (MIMO) system for Simultaneous Wireless Information and Power Transfer (SWIPT).

The phenomenon of simultaneous wireless information and power transfer was derived from the idea that RF signals can carry energy that is used for transmitting the information [91]. SWIPT has been identified as a sustainable proposition for harvesting of energy from the radio frequency (RF) signals, which in turn is supplied to finite-powered wireless communication devices including wireless sensors and electronic gadgets [92,93]. As it can be seen in Figure 17, the transmitter transmits the information signal to the information receivers and also transfers the power to the energy receivers responsible for harvesting the energy [94]. However, it has been shown that in some cases the energy-harvesting receivers might have a better channel for receiving the information aimed at the information receivers, and they might jeopardize the security of the transmitted information. Additionally, efficient methods are also required for improving the efficiency of energy-harvesting receivers to enhance the amount of energy that can be harvested. In order to address these challenges, PLS has been employed on SWIPT in many different studies [92,93,94,95,96,97,98,99]. The authors of [94] investigated SWIPT for MISO secrecy channel and considered transmit beamforming for two cases, with AN and without AN, with the aim of maximizing the secrecy rate as well as the harvested energy. Boshkovska et al. in [93] proposed a robust resource allocation scheme jointly responsible for time allocation and power control, taking into consideration uncertainty regarding the CSI. In [100], the authors consider a secure beamforming design for SWIPT in heterogeneous cellular networks which are formed by a single macrocell consisting of multiple macrocell users and a single femtocell comprising a single information receiver and multiple eavesdroppers. They use semi-definite programming and proposed an iterative algorithm to maximize the secrecy rate at information receivers while guaranteeing the SINR requirement recorded at each macrocell user.

#### 4.2.2. Machine Learning (ML)-Based Channel Estimation

The development of channel estimation techniques has been investigated in [101,102,103,104]. From these investigations it is evident that tackling imperfect CSI in PLS is one of the problems still open for research. The conventional channel estimation methods based on channel modeling have been proved to be insufficient for providing accurate and timely CSI. There has been a recent surge in research directed towards the feasibility of tackling some of the various communication problems using ML [101]. Most of the research is devoted towards developing efficient and reliable algorithms for channel estimation in communication networks. The performance of the existing channel estimation algorithms can be augmented through the use of ML to achieve close-to-optimal algorithms with reduced complexity on the implementation [47]. ML has emerged as an effective tool for channel estimation in wireless communication systems, especially under some imperfect environments.

#### 4.2.3. Unmanned Aerial Vehicle (UAV)

**Figure 18:**
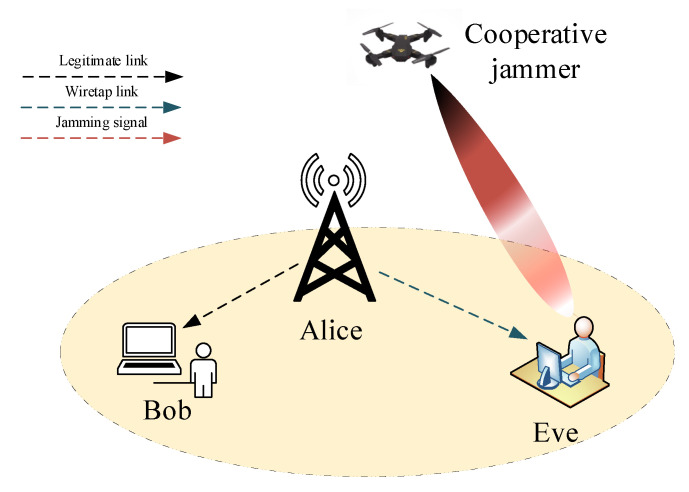
Cooperative jamming using unmanned aerial vehicle (UAV).

Drones or UAV-based communication technology has been thoroughly studied and adopted by the 3GPP standard [105]. A study on UAV systems that highlighted and gave an overview of the latest advances and current state of research in the field of PLS was conducted [105]. UAV systems have been envisaged to form an integral part of future wireless communication applications due their dynamic, flexible, and flying nature. It was shown in [106] that, due to their ability to reach higher altitudes, they usually have dominant LOS channels with the ground nodes. This capability can be used to provide confidentiality to the legitimate receivers against the eavesdroppers. This can be done by deploying UAVs to launch more effective jamming signal attacks to terrestrial eavesdroppers, as shown in Figure 18. The conventional cooperative jamming schemes make an assumption that the locations of terrestrial jammers are fixed, which might compromise the secrecy of the system if the jammers are located far away from the eavesdroppers, and is also not practical as it makes an assumption of perfect CSI of the jammer to eavesdropper channel [107]. Authors in [107] deployed a UAV-based system as a jammer to improve the secrecy rate of a ground wiretap channel. Wu et al. [15] considered a scenario in which a UAV is equipped with an air-to-ground jammer and a ground communication network comprising a legitimate transmitter–receiver pair and an eavesdropper. They proposed an iterative algorithm to maximize the achievable average secrecy rate of a wireless communication system.

#### 4.2.4. Intelligent Reflecting Surface (IRS)

Future wireless networks are expected to employ intelligent and software re-configurable functionalities to enable safe and secure communication [108]. Intelligent reflective surfaces have been identified as a solution to create a controllable wireless environment. According to [109], an IRS is a software-controlled artificial surface that can be programmed to alter its electromagnetic response. An IRS can change the attenuation and scattering of the incident electromagnetic wave so that it can propagate in the desired way towards the intended receiver by adjusting the reflecting coefficients. Authors in [110] discussed two methods of deploying IRS, namely (a) energy focusing and (b) energy nulling. Energy focusing employs a beamforming technique by using the IRS reflecting elements to adjust the phases of the signal coming from the transmitter, so that it is focused towards one intended user. Meanwhile, energy nulling uses the IRS to perform destructive reflection by adjusting the phases of the scattered signals to null out the signal at the unintended recipients [110]. One application of IRS is in holographic beamforming, as shown in [111]. Holographic beamforming is a dynamic beamforming technique that uses a software-defined antenna (SDA).

To demonstrate the application of IRS in PLS, we consider a communication setup of a MISO shown in Figure 19 from [14]. The system comprises a single transmitter, Alice, with *N* antennas; an intended receiver, Bob; and one eavesdropper, Eve. Both Bob and Eve have single receive antennas. The system is also made of an IRS which has L reflecting elements. The received signals at Bob and Even are given by

(37) $$y_{B}=h_{IB}^{H}\Theta H_{AI}x+h_{AB}^{H}x+n_{B}$$

(38) $$y_{E}=h_{IE}^{H}\Theta H_{AI}x+h_{AE}^{H}x+n_{E}$$

where $h_{IB}$ is the IRS–Bob channel, $\Theta =\mathrm{diag}\{\left[e^{\left(j\phi_{1}\right)},\ldots ,e^{\left(j\phi_{i}\right)},\ldots ,e^{\left(j\phi_{L}\right)}\right]\}$ is the diagonal with $\phi_{i}$ denoting the phase shift of the *i*-th reflecting element of the IRS, $H_{AI}$ is the Alice–IRS channel, x is the transmitted signal with maximum transmit power, *P*, $h_{AB}^{H}$ is the Alice–Bob channel, and $n_{B}$ is the AWGN noise at Bob with variance $\sigma_{n,B}^{2}$. Similarly, subscript ${\left\{\cdot \right\}}_{E}$ denotes parameters with relevance to Eve.

The achievable secrecy rate is given by

(39) $$\begin{array}{cc} R_{s} & =R_{b}-R_{e}\\ \; & =\log_{2}\left(1+\frac{h_{IB}^{H}\Theta H_{AI}h_{AB}^{H}PH_{AI}^{H}\Theta^{H}h_{IB}+h_{AB}}{\sigma_{n,B}^{2}}\right)\\ \; & -\log_{2}\left(1+\frac{h_{IE}^{H}\Theta H_{AI}h_{AE}^{H}PH_{AI}^{H}\Theta^{H}h_{IE}+h_{AE}}{\sigma_{n,E}^{2}}\right) \end{array}$$

**Figure 20:**
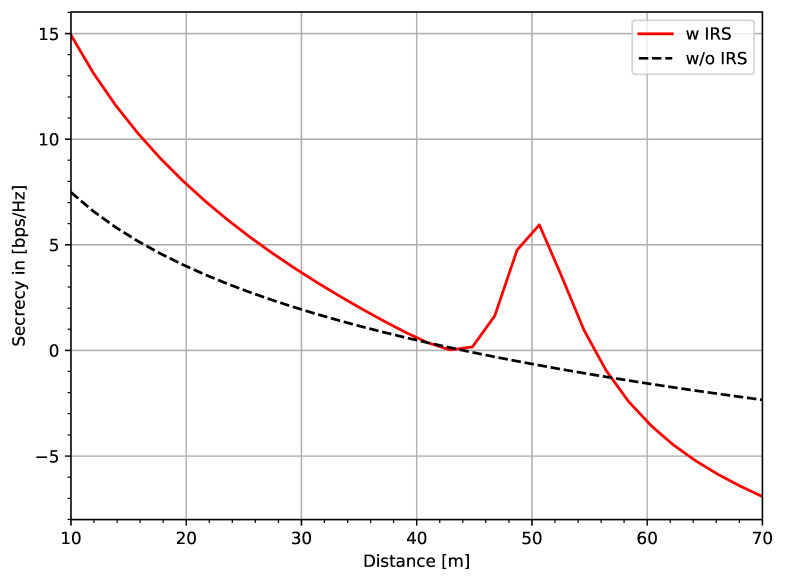
Secrecy rate vs distance.

The performance of the secrecy rate in Equation (39) is illustrated by the simulation shown in Figure 20. The secrecy rate is shown by varying the distance between Alice and Bob. Figure 20 clearly shows that the performance of a PLS scheme without an IRS yields a lower secrecy rate with an increasing distance between Bob and Alice. On the other hand, an improved secrecy rate can be achieved when IRS is introduced, as shown in the above-mentioned figure. However, it should be noted that optimization techniques may significantly and efficiently enhance the secrecy rate, as shown in [14].

#### 4.2.5. Visible Light Communication (VLC)

**Figure 21:**
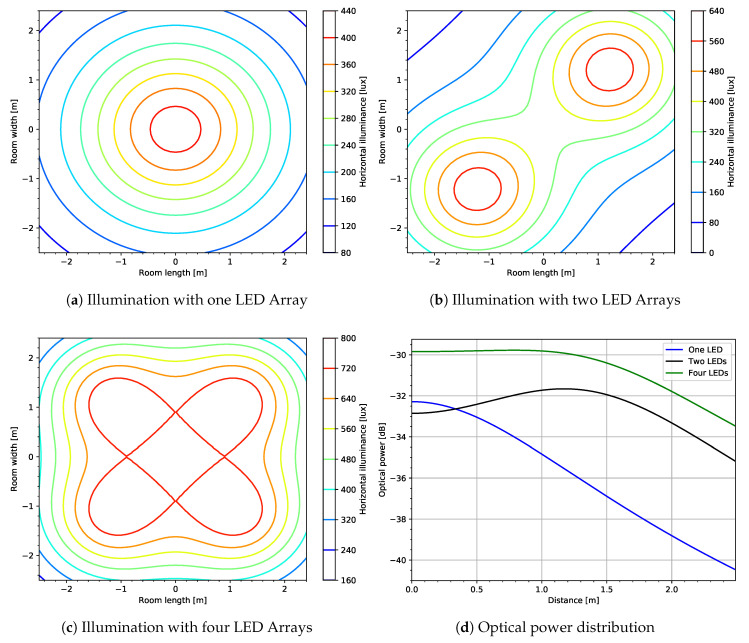
Power and illumination distribution vs distance.

VLC is one of the technologies which has emerged with the potential of providing omnipresent access to wireless broadband for indoor settings [10,112]. This is enabled by some of its unique characteristics, which include line-of-sight propagation, inability of light waves to penetrate opaque surface, technological advancements, and economical costs of lighting equipment such as solid-state light-emitting diodes (LEDs). The aforementioned characteristics are highly useful in PLS since VLC signals are confined inside the building’s walls, hence an inherently secure communication which prevents eavesdropping for outdoor eavesdroppers. VLC schemes are realized through the use of a number of LED arrays and photodetectors (PDs). The LED arrays serve a dual purpose of offering illumination and data transmission simultaneously while the PDs are used as receivers. The most common method of modulation in VLC is the intensity modulation and direct detection (IM/DD). Unlike in RF systems, the modulating signals in VLC systems must have real, non-negative values [113]. To show the concept of VLC, Figure 21 depicts the illumination and power distribution using various arrays made up of red-green-blue (RGB) LEDs.

Horizontal illuminance of a light fixture made up of an LED array of 50×50 RGB LED chips with a semi-angle of 60° is depicted in Figure 21a. Each LED chip has a Lambertian radiation pattern and total luminous intensity of 33.74cd. As seen in Figure 21b, increasing the number of LED arrays leads to an increased intensity and luminescence at all points of the room. Consequently, this results in elevated optical power, as seen in Figure 21d. Optical power is crucial for data transmission because direct detection depends on the amount of power which impinges the PD. Due to incoherently distributed light intensity, the amount of optical power produced by the LED arrays decreases logarithmically with increasing distance.

#### 4.2.6. PLS in Satellite Communication

**Figure 22:**
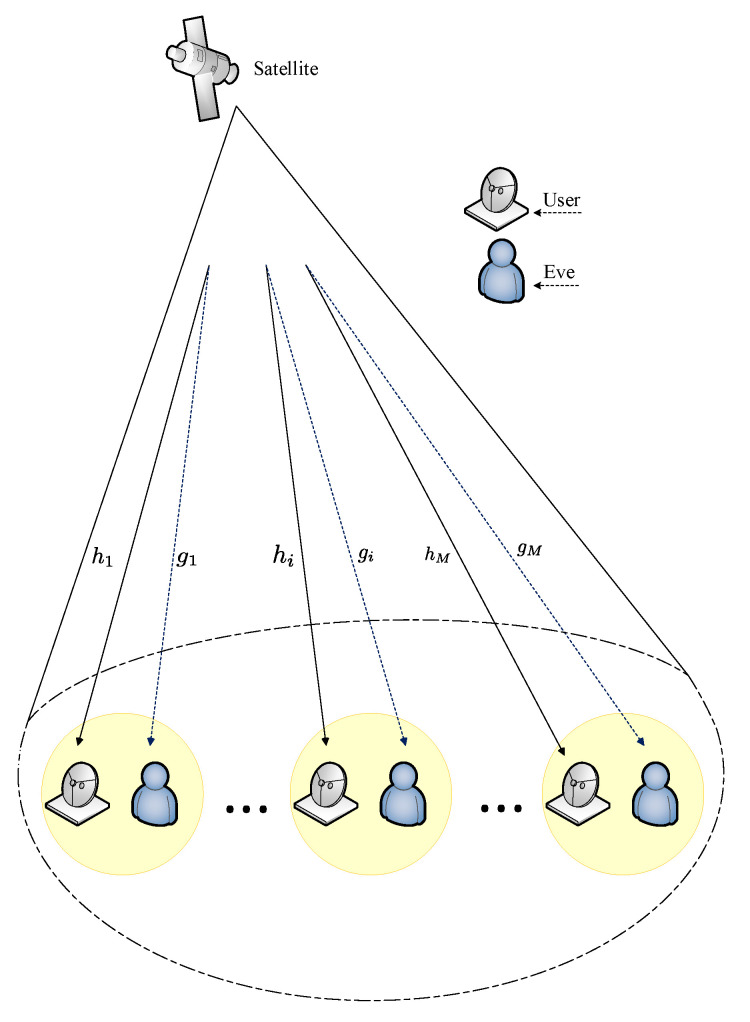
Multi-beam satellite communication network [114].

Satellite communication is being more incorporated into current terrestrial wireless communication networks mainly because of its features, which include wide-area coverage and high bandwidth. Some noteworthy applications of satellite communication include, but are not limited to, military operations, TV broadcast, and internet access. However, their broadcast nature and ability to provide wide coverage area make them more vulnerable to eavesdropping. Traditionally, security in satellite communication is implemented in the upper layers of the protocol stack by means of encryption. PLS has been identified as an alternative to augment the current cryptography security measures [21]. We demonstrate a PLS concept in satellite using a system model shown in Figure 22 and derive the optimization problem.

Suppose that the satellite transmits the signal $s_{i}$ intended for the *i*th legitimate user with average power $E[{\left|s_{i}\right|}^{2}]=1$. The transmitted signal has a weighted beamforming vector given by $w_{i}\in C^{N\times 1}$. Therefore, the overall transmitted signal is given by

(40) $$x=\sum_{i=1}^{M}w_{i}s_{i}$$

The signals received by the *i*th legitimate and *i*th eavesdropper are given by Equations (41) and (42), respectively.

(41) $$y_{user-i}=h_{i}^{H}w_{i}s_{i}+\sum_{m\neq i}^{M}h_{i}^{H}w_{m}s_{m}+n_{i}$$

(42) $$y_{e-i}=g_{i}^{H}w_{i}s_{i}+\sum_{m\neq i}^{M}g_{i}^{H}w_{m}s_{m}+n_{e-i}$$

where $h_{i}\in C^{N\times 1}$ denotes the channel gain vector between the *i*th intended user and the satellite, $g_{i}\in C^{N\times 1}$ denotes the channel gain vector between the *i*th eavesdropper and the satellite, and $n_{i}$ and $n_{e-i}$ are assumed to be zero-mean AWG noise of the *i*th intended user and the *i*th eavesdropper, respectively. The achievable secrecy rate of the *i*th intended user is given by

(43) $$R_{s}=\log_{2}\left(1+\frac{{\left|h_{i}^{H}w_{i}\right|}^{2}}{\sum_{m\neq i}^{M}{\left|h_{i}^{H}w_{m}\right|}^{2}+\sigma_{i}^{2}}\right)-\log_{2}\left(1+\frac{{\left|g_{i}^{H}w_{i}\right|}^{2}}{\sum_{m\neq i}^{M}{\left|g_{i}^{H}w_{m}\right|}^{2}+\sigma_{e-i}^{2}}\right)$$

The typical optimization problem of interest is to maximize the achievable secrecy rate and can be written mathematically as follows:

(44) $$\begin{array}{cc} \min_{w_{i}}\; & R_{s}\\ s.t.\; & \sum_{m=1}^{M}{\left|\left|w_{i}\right|\right|}^{2}\leq P_{T} \end{array}$$

where $P_{T}$ is the maximum transmit power of the satellite. The authors in [115] studied the problem of minimizing the transmit power on a multi-beam satellite while fulfilling the minimum per user secrecy rate. They further proposed an iterative algorithm to jointly optimize the transmission power and the beamforming vector by completely eliminating the co-channel interference and perfectly nulling out the received signal at the eavesdropper. Kalantari et al. in [116] also used PLS to address the issue of confidentiality in bidirectional satellite communication based on network coding. They designed the optimal beamforming weight vector which maximizes the sum secrecy rate by using semi-definite programming.

## 5. Conclusions

We have provided a comprehensive review of PLS in wireless networks based on optimization techniques. We have also shown that PLS is an auspicious technology for strengthening the confidentiality and secrecy of information transmission in both existing and emerging wireless networks, which can be used to augment conventional cryptographic methods. To emphasize the benefits of PLS, we first compared conventional encryption using cryptography and PLS. This work mainly focused on the provision of a comprehensive review of both the design and the optimization of PLS schemes. We also discussed some of the main challenges facing PLS and outlined some of the promising solutions to these problems and how they can benefit future wireless communication networks. In short, we have shown that PLS is a very promising technology in ensuring safe and secure wireless communication.

## Figures and Tables

**Figure 19:**
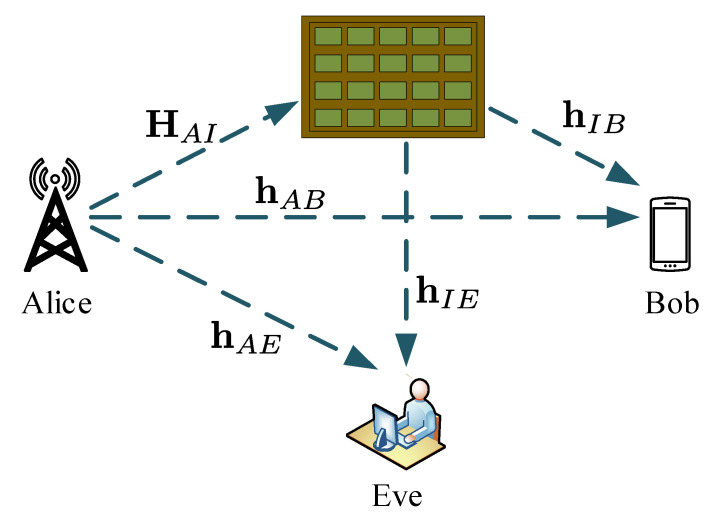
Intelligent Reflecting Surface (IRS) system model [14].

## Abbreviations

The following abbreviations are used in this manuscript:

AN	Artificial noise
AWGN	Additive white Gaussian noise
BER	Bit error rate
CSI	Channel state information
IRS	Intelligent reflecting surface
ITU	International telecommunication Union
MIMO	Multiple-input multiple-output
OFDM	Orthogonal frequency division multiplexing
PLS	Physical layer security
QoS	Quality of service
SINR	Signal-to-interference-plus-noise ratio
STBC	Space-time block code
UAV	Unmanned aerial vehicle
URLLC	Ultra-reliable low latency communication
WIPT	Wireless information and power transfer
ZF	Zero-forcing
